# Assessment of interfractional variation of the breast surface following conventional patient positioning for whole‐breast radiotherapy

**DOI:** 10.1120/jacmp.v15i5.4921

**Published:** 2014-09-08

**Authors:** Laura Padilla, Hyejoo Kang, Maxine Washington, Yasmin Hasan, Steve J. Chmura, Hania Al‐Hallaq

**Affiliations:** ^1^ Department of Radiation and Cellular Oncology University of Chicago Chicago IL USA

**Keywords:** WBRT, surface imaging, patient positioning, MV portal films

## Abstract

The purpose of this study was to quantify the variability of the breast surface position when aligning whole‐breast patients to bony landmarks based on MV portal films or skin marks alone. Surface imaging was used to assess the breast surface position of 11 whole‐breast radiotherapy patients, but was not used for patient positioning. On filmed fractions, AlignRT v5.0 was used to capture the patient's surface after initial positioning based on skin marks (28 “preshifts” surfaces), and after treatment couch shifts based on MV films (41 “postshifts” surfaces). Translations and rotations based on surface captures were recorded, as well as couch shifts based on MV films. For nonfilmed treatments, “daily” surface images were captured following positioning to skin marks alone. Group mean and systematic and random errors were calculated for all datasets. Pearson correlation coefficients, setup margins, and 95% limits of agreement (LOA) were calculated for preshifts translations and MV film shifts. LOA between postshifts surfaces and the filmed treatment positions were also computed. All the surface captures collected were retrospectively compared to both a DICOM reference surface created from the planning CT and to an AlignRT reference surface. All statistical analyses were performed using the DICOM reference surface dataset. AlignRT reference surface data was only used to calculate the LOA with the DICOM reference data. This helped assess any outcome differences between both reference surfaces. Setup margins for preshifts surfaces and MV films range between 8.3–12.0 mm and 5.4–13.4 mm, respectively. The largest margin is along the left–right (LR) direction for preshift surfaces, and along craniocaudal (CC) for films. LOA ranges between the preshifts surfaces and MV film shifts are large (12.6–21.9 mm); these decrease for postshifts surfaces (9.8–18.4 mm), but still show significant disagreements between the two modalities due to their focus on different anatomical landmarks (patient's topography versus bony anatomy). Pearson's correlation coefficients further support this by showing low to moderate correlations in the anterior–posterior (AP) and LR directions (0.47–0.69) and no correlation along CC(<0.15). The use of an AlignRT reference surface compared to the DICOM reference surface does not significantly affect the LOA. Alignment of breast patients based solely on bony alignment may lead to interfractional inconsistencies in the breast surface position. The use of surface imaging tools highlights these discrepancies, and allows the radiation oncology team to better assess the possible effects on treatment quality.

PACS number: 87

## I. INTRODUCTION

Accurate and consistent patient positioning is crucial for ensuring the successful delivery of any radiation therapy treatment. Anatomical regions with high tissue deformability, such as the breast, are especially challenging to position reproducibly. Thus, breast positioning, image guidance, and clinical outcome are areas of ongoing study.^1^, ^2^, ^3^ Typically, daily alignment is performed by verifying the light field projection or the source‐to‐skin distance (SSD) on the patient. Weekly verification of positioning relies on the use of MV or kV X‐ray portal images to match bony landmarks. However, these standard techniques may lead to clinically significant inter‐ and intrafraction positional variability for some individuals.^(2)^ Attempts to improve alignment procedures focusing on bony anatomy do not consider the breast tissue, which may deform and change volume throughout treatment. As a result, deformation can cause the tissue encompassed by the planning target volume (PTV) to vary. Jain et al.^(4)^ investigated the effect of these changes on the PTV and its corresponding dosimetry delivered by intensity‐modulated tangential fields. This study concluded that current positioning and verification methods lead to significant interfraction breast variability that dosimetrically degrades the treatment delivery. As breast treatments become more localized (i.e., partial breast irradiation) and/or more conformal (i.e., inverse‐planned IMRT), the discrepancy between bony versus soft tissue alignment could have a larger effect on accurate dose delivery.^(2)^ These data demonstrate the need for improvements in intra‐ and interfraction monitoring of the breast tissue itself.

The use of surface imaging to assess external topography for breast radiotherapy could improve the reproducibility and accuracy of patient positioning. Several publications have reported the potential benefit of surface imaging as an additional tool for patient positioning for various breast irradiation techniques.^5^, ^6^, ^7^ Some institutions use surface imaging to gain a dosimetric advantage by implementing deep‐inspiration breath‐hold radiotherapy to separate left‐sided breast cancer targets from the heart.^8^, ^9^, ^10^ Because surface imaging utilizes the breast surface itself for positioning, it may provide greater soft tissue reproducibility than X‐ray imaging. Furthermore, surface imaging may be more sensitive to changes in the breast surface shape or volume. If these breast changes are significant and nontransient over the duration of the treatment course, they could systematically alter the dose delivered to the target. The use of surface imaging could highlight these occurrences and provide the treatment team with additional information on the shape and position of the patient's breast in real time.

The purpose of this study is to use surface imaging to quantify the positional variation of the breast surface when bony anatomy alignment, as quantified by comparison to the simulated position, is utilized for treatment setup. This will provide important information on whether the use of alternative positioning tools, like surface imaging, is warranted when the specific target of interest is highly deformable, such as breast tissue, and its position cannot be reliably correlated to bony landmarks.

## II. MATERIALS AND METHODS

AlignRT v5.0 (VisionRT, London, UK) with a three‐camera installation was used to assess the position of 11 patients undergoing tangential whole‐breast irradiation. The data were collected following an IRB‐approved protocol. The goal of this study was not to position patients using AlignRT, but to use this technology to quantify the level of agreement between the “ideal” treatment positions according to MV films versus surface imaging. This is a retrospective study and, although surface imaging was performed during treatment fractions, the information was not used for positioning patients. Table 1 summarizes the characteristics of the patients enrolled in this study.

AlignRT assesses real‐time patient positioning by comparison to a reference surface. This reference surface is user‐defined and can be imported from a DICOM file or created from a surface recorded using AlignRT. The software registers the real‐time surface to the reference and outputs the difference in position as a “delta” value in millimeters for three translations, representing anterior–posterior (AP), craniocaudal (CC), left–right (LR), and in degrees for three rotations (roll, pitch, yaw). It uses a rigid body iterative closest‐point (ICP) algorithm to determine the dimensions of the translations and rotations necessary to make the current surface match the reference.^(11)^ Delta values were collected and analyzed for each patient. Only surfaces that were complete, in which the gantry or imaging arms did not obscure the surface projection, were included in the analysis.

**Table 1:** Patient characteristics

*Characteristics*	*No. of Patients (if applicable)*
Number of patients	11
Age (y)	
Median (range)	59 (41‐81)
T stage	
Tis	1
T1	8
T2	2
N stage	
Nx	1
N0	9
N1(mic) or N1	1
Treatment site	
Left breast	6
Right breast	5
Number of fractions	
Median (range)	25 (16,28)
Body mass index (kg/m^2^)	
Median (range)	29 (21‐36)
18.5‐25 (normal)	3
>25‐30 (overweight)	4
>30 (obese)	4

### A. Data collection

All patients receiving whole‐breast radiation, typically delivered four to six weeks postsurgery, were sequentially enrolled onto the study. All patients were positioned on a C‐Qual slant board (CIVCO Medical Solutions, Orange City, IA) with both arms above the head and immobilized with a customized upper alpha cradle. According to our institution's routine clinical protocol, patients were positioned to three‐point alignment marks, shifted to the isocenter position where alignment was further verified and refined based on light field projection of the tangent fields onto the patient's lateral and medial entry marks. Filming verification began with alignment based upon an orthogonal AP and LR pair, followed by refinement to the MV tangential portal films. Skin marks on the patient were updated following each filming session.

**Figure 1:**
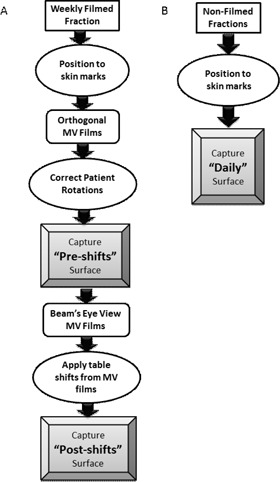
Charts depicting the workflow of patient positioning and surface image captures for weekly filmed fractions (a), and nonfilmed fractions (b).

The flow chart shown in Fig. 1 summarizes the workflow used to acquire the surface images in this study. For filmed fractions acquired weekly, an AlignRT surface was recorded after positional correction for rotations, but before correction for translations based on films (i.e., after patient rotations were made, but before table shifts were applied). This will be referred to as the “preshifts” surface. After translating the couch position according to MV films, a “postshifts” surface was recorded. This surface characterizes the discrepancies between the planned position and the treatment position determined with MV films. Thus, the differences between “preshifts” and “postshifts” surfaces were simply translational and could be directly compared to translations made with MV filming. We chose to focus on translations only because rotational corrections from orthogonal films could not be quantified and directly compared to those calculated by AlignRT.

On nonfilm days, a “daily” surface was captured after the patient was aligned according to skin marks, but prior to delivering the treatment. These data were collected to assess daily variations in patient positioning and the breast surface with current alignment methods (i.e., skin marks alone). AlignRT was not used to position patients for any of the treatments. Since none of the treatments were gated, surface captures were not gated either.

Our goal was to determine how well the patient's position at simulation was reproduced throughout treatment. Thus, a reference surface, ${\text{DICOM}}_{\text{ref}}$, was created from the external surface of the treatment planning CT scan. The scan was acquired with free‐breathing on a Phillips Brilliance Big Bore CT scanner (Philips Healthcare, Andover, MA) using 3 mm slice thickness with pixel dimensions ranging from 1.07–1.37 mm. The external surface was automatically contoured using a density threshold of 0.6g/cm^3^ in Pinnacle v9.0. To determine if there is an advantage to using a reference surface acquired with the AlignRT cameras, a second reference surface was used for analysis. An AlignRT reference surface (${\text{AlignRT}}_{\text{ref}}$) was retrospectively created for each patient using the “postshifts” capture that best matched the simulated position (i.e., best registered to DICOM surface). Ideally, ${\text{AlignRT}}_{\text{ref}}$ should be acquired during the time of CT simulation. However, surface imaging cameras were not available in our simulator.

**Figure 2:**
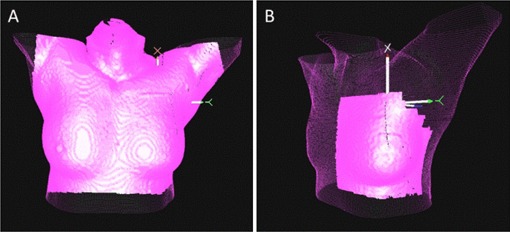
AlignRT regions of interest used for data acquisition in the study: (a) entire surface; (b) breast.

The delta values calculated by AlignRT are based upon registration of regions of interest (ROI) defined by the user on the reference surface. For this study, the ROIs selected were the ‘entire’ surface of the patient from the bottom of the mandible to the umbilicus and the treated ‘breast’ surface, as illustrated in Fig. 2. The entire surface ROI selection was utilized to better assess patient rotations that would be difficult to discern using a smaller ROI. The breast surface ROI was selected to evaluate the position of the specific treatment area. The pendulous tissue was not omitted from this selection because the assessment of its positional variation was of interest for this study. The deltas for 28 preshift surfaces, 41 postshift surfaces, and 162 daily surfaces were collected and analyzed for both the breast and entire surface ROIs. The discrepancy in the number of preshift surfaces and postshift surfaces is due to inconsistencies in surface recording during the 41 filmed fractions included in this study. All available complete surfaces were used for analysis.

### B. Statistical analysis

Several statistical parameters were calculated to compare the shifts output by registration to both ${\text{AlignRT}}_{\text{ref}}$ and ${\text{DICOM}}_{\text{ref}}$ to those resulting from MV filming. The group mean, systematic error, and random error for MV films and AlignRT datasets (preshift, postshift) were calculated for all translations. The systematic and random errors were then used to calculate the required setup margins based on the van Herk formulation^(12)^ (see Eq. (1)). Setup margin allows for a direct comparison between AlignRT and MV positioning because it combines the effects of systematic and random errors into a single value.

(1) Δ=2.5∑+0.7σ

where Δ is the setup margin, Σ is the systematic error, and *σ* is the random error. All quantities are in millimeters.

Pearson correlation coefficients for AlignRT deltas versus MV film shifts along all translational directions were also computed. The 95% limits of agreement (LOA) between preshift deltas and MV films shifts were calculated (Eq. (2)) to quantify the range of the discrepancies between translations suggested by AlignRT and MV films. The LOA between postshift surfaces and the treatment position according to MV films (i.e., AP=0mm,CC=0mm,LR=0mm) were also calculated. These values indicate the magnitude of difference between surface imaging and the treatment position as verified by MV imaging. LOAs were calculated for ${\text{AlignRT}}_{\text{ref}}$ data, as well.

(2) LOA=μ±1.96*σ

here *μ* represents the mean difference (bias) and *σ* is the standard deviation. If the limits of agreement are asymmetric, this indicates that the mean is nonzero and there is a bias in the results. Box plots of the daily surface data using both ${\text{DICOM}}_{\text{ref}}$ and ${\text{AlignRT}}_{\text{ref}}$ were constructed defining the 25th, median, and 75th percentile values for the distributions.

### C. MV films to DRR surface comparison

Each MV tangent film acquired at the final treatment position was retrospectively visually inspected to compare the coincidence of the external surface to the outline of the digitally reconstructed radiographs (DRRs). These images were used to provide a qualitative visual check of the breast surface discrepancy when bony anatomy is used to determine a patient's treatment position. Since each patient had several filmed fractions, it allowed intrapatient surface variations throughout the course of treatment to be recorded. The window and level of each image was adjusted to properly display the entire breast surface on each film.

## III. RESULTS

### A. Statistical analysis

Table 2 presents the statistical analysis results from the ${\text{DICOM}}_{\text{ref}}$ data. For MV films, the setup margin in the AP direction is less than half the magnitude of setup margins in the CC and LR directions. Surface imaging setup margins calculated for the preshifts results are comparable to MV films along the LR direction (0.1–1.2 mm), smaller by 3.6–5.1 mm along the CC direction, and larger by 3.8–4.8 mm along the AP direction. The exact values depend on the ROI used for registration. The correlation coefficient results show that the lowest correlation (r<0.3) is consistently found in the CC direction and the highest correlation (r=0.66‐0.69) is in the LR direction, for both ROIs.

**Table 2:** Statistical analysis results for MV films and surface imaging (SI) data using the DICOM reference surface (${\text{DICOM}}_{\text{ref}}$) for preshifts and postshifts surfaces along the anterior‐posterior (AP), craniocaudal (CC), and left‐right (LR) directions

	*Preshifts (mm)*			*Postshifts (mm)*		
	*AP*	*CC*	*LR*	*AP*	*CC*	*LR*
SI ‐ Entire Surface						
Group mean	0.9	3.8	2.2	0.6	1.9	0.3
Systematic error	3.3	3.0	4.0	2.2	2.4	3.1
Random error	2.6	3.4	3.0	1.8	2.6	2.7
LOA: upper bound	9.0	14.1	7.9	6.2	9.3	7.9
LOA: lower bound	‐3.9	‐7.0	‐7.6	‐3.6	‐4.3	‐6.9
LOA range	12.8	21.1	15.5	9.8	13.6	14.8
Setup margin	10.2	9.8	12.0	‐	‐	‐
r	0.49	0.14	0.66	‐	‐	‐
SI ‐ Breast Surface						
Group mean	0.3	1.2	3.4	‐0.7	‐1.3	1.7
Systematic error	3.1	2.5	3.0	2.8	3.2	3.1
Random error	2.3	2.9	4.8	2.1	3.0	2.8
LOA: upper bound	8.3	11.2	8.8	6.4	8.3	9.9
LOA: lower bound	‐4.3	‐10.7	‐6.3	‐6.6	‐10.1	‐6.1
LOA range	12.6	21.9	15.1	13.0	18.4	15.9
Setup margin	9.2	8.3	10.9	‐	‐	‐
r	0.47	‐0.07	0.69	‐	‐	‐
MV Films						
Group mean	‐1.0	‐0.1	2.7	‐	‐	‐
Systematic error	1.5	4.8	3.8	‐	‐	‐
Random error	2.5	1.8	3.5	‐	‐	‐
Setup margin	5.4	13.4	12.1	‐	‐	‐

Table 3 compares the LOA calculated for both ${\text{AlignRT}}_{\text{ref}}$ and ${\text{DICOM}}_{\text{ref}}$. The LOA ranges between the MV film and preshifts surfaces are greater than 10 mm regardless of the ROI or reference surface used. For both ROIs and reference surfaces, the preshifts LOA ranges are consistently largest along the CC direction and smallest along the AP direction. The postshift LOAs are reduced for both reference surfaces. While there is some variability of up to 1–4 mm, depending upon the reference and ROI, LOA ranges are comparable for both reference surfaces for each ROI and translational direction. The LOA results indicate that, overall, there is a bias between the shifts indicated by AlignRT and MV films, irrespective of statistical fluctuations.

**Figure 3:**
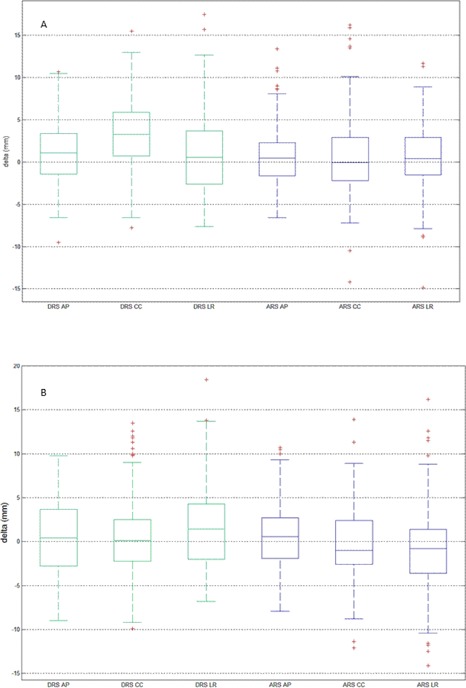
Box plots of the daily surface data for all patients (n=162) for the entire surface ROI (a) and the breast surface ROI (b) (DRS=DICOM Reference Surface,ARS=AlignRT Reference Surface). Box lines show the 25th, median, and 75th percentile values. The bars indicate the smallest and largest nonoutlier values. Red crosses designate outliers that are values beyond 1.5 (indicated by astericks) interquartile range from the 25th and 75th percentile values.

Figure 3 shows the boxplots for the entire (a) and breast (b) surface ROIs for the daily data collected from all patients. For the entire surface ROI, median values are closer to 0 across all directions for ${\text{AlignRT}}_{\text{ref}}$ than ${\text{DICOM}}_{\text{ref}}$. This does not hold true for the breast surface ROI data, as the median values along AP and CC are closer to 0 with ${\text{DICOM}}_{\text{ref}}$ than ${\text{AlignRT}}_{\text{ref}}$. Median values are furthest from zero along the CC direction for the entire surface ROI using ${\text{DICOM}}_{\text{ref}}$. The median values are overall closer to zero for all ROIs when using ${\text{AlignRT}}_{\text{ref}}$. The interquartile range, represented by the length of the box, is comparable along the CC and LR directions for both ROIs using ${\text{DICOM}}_{\text{ref}}$, but noticeably larger along AP for the breast surface ROI. The ${\text{AlignRT}}_{\text{ref}}$ data have smaller interquartile ranges than ${\text{DICOM}}_{\text{ref}}$ in all cases, except for the breast ROI along the AP direction, but it has an overall larger percentage of outliers.

The mean±standard deviation of all rotations for the daily datasets were calculated. The yaw, roll, and pitch calculated using ${\text{DICOM}}_{\text{ref}}$ are larger for the breast surface ROI(0.2°±1.7°,0.5°±1.6°,‐0.6°±1.3°) than for the entire surface ROI(‐0.4°±1.3°,‐0.5°±0.7°,‐0.5°±0.9°). While the rotational magnitudes decrease when calculated using ${\text{AlignRT}}_{\text{ref}}$, they remain larger for the breast surface ROI(‐0.5°±1.4°,0.2°±1.1°,‐0.3°±1.0°) compared to the entire surface ROI(‐0.1°±0.9°,0.0°±0.7°,‐0.3°±0.9°).

**Table 3:** Limits of agreement between the AlignRT deltas and the shifts indicated by MV films (preshifts) and limits of agreement between AlignRT deltas of the surface treatment position and the actual treatment position (postshifts)

	*Preshifts (mm)*			*Postshifts (mm)*		
	*AP*	*CC*	*LR*	*AP*	*CC*	*LR*
${\text{DICOM}}_{\text{ref}}$						
Entire Surface	12.8	21.1	15.5	9.8	13.6	14.8
Breast Surface	12.6	21.9	15.1	13.0	18.4	15.9
${\text{AlignRT}}_{\text{ref}}$						
Entire Surface	12.6	25.2	16.3	8.5	14.9	11.7
Breast Surface	12.4	20.6	19.1	12.0	15.8	14.9

### B. MV films to DRR surface comparison

**Figure 4:**
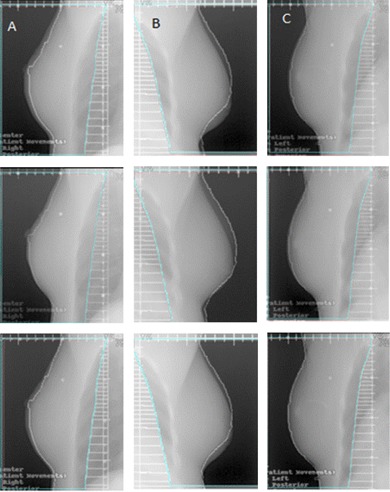
Digitally reconstructed radiograph and MV film comparison for three different patients (Columns A, B, and C). The interpatient variation is shown across the rows, while the intrapatient variation is shown down each column. Each tick‐mark on the axis represents 1 cm.

Figure 4 shows the comparison between the DRR and MV film surfaces for three patients during three different filmed fractions. The white outline represents the external surface of the DRR (i.e., the projection of ${\text{DICOM}}_{\text{ref}})$. The MV images shown were acquired in the treated position. Across each row the interpatient variation can be observed, while down each column the intrapatient variation is illustrated. Patient A shows a consistent directional offset between the MV and DRR surfaces; the magnitude of the offset varies between fractions. Patient B shows more variation in the relative position of the MV and DRR surfaces. For this patient, the breast center of mass is more caudal than planned. Patient C shows a consistent setup with respect to the DRR and throughout all filmed fractions. Table 4 shows the postshift surface means and standard deviations for the patients featured in Fig. 4. The breast surface has a CC offset (7.4 mm) for Patient B and a LR offset (5.1 mm) for Patient A. Overall, Patient B has the largest means and standard deviations, indicating that the external surface mismatch was large and highly variable throughout the treatment course. Patient A has a higher mean magnitude and larger standard deviations compared to Patient C. This indicates that Patient A had higher interfraction variability of the breast surface, while Patient C had a more consistent setup throughout treatment.

**Table 4:** Postshifts means and standard deviations (SDs) for the patients shown in Fig. 4 along the anterior–posterior (AP), craniocaudal (CC), and left–right (LR) directions

		Mean±SD(mm)	
		*Entire*	*Breast*
Patient A	AP	2.5±2.1	0.6±1.7
	CC	1.4±3.0	‐0.2±4.4
	LR	1.9±1.6	5.1±1.4
Patient B	AP	3.3±2.8	3.7±2.4
	CC	5.9±2.3	7.4±1.7
	LR	‐2.0±2.5	‐0.8±3.6
Patient C	AP	‐3.5±0.6	‐2.0±0.7
	CC	‐2.8±1.9	‐2.3±0.6
	LR	‐2.7±0.6	0.6±1.2

## IV. DISCUSSION

Analysis of the uncorrected patient positions shows that setup margins for surface imaging and MV films are comparable (∼10mm across all directions). This implies that both modalities would provide similar reproducibility of patient positioning compared to alignment of skin marks alone. It is important to emphasize that AlignRT was not used to adjust patient positions. Consequently, setup margins calculated for surface imaging might be overestimated. If AlignRT were used to position the patient, correction of small patient rotations detected by the software, calculated to be <1° on average, could have affected the registration algorithm, thereby decreasing translational deltas.

Comparison of the preshift surfaces to MV film positions demonstrate a large discrepancy between the shifts identified by AlignRT and films (LOA ranges >12mm). This is expected since each modality uses different anatomical structures (i.e., bony anatomy versus skin surface) to determine the ideal treatment position. This is confirmed by the low correlation coefficients between the shifts indicated by the two modalities, which is lowest in the CC direction. Treatment position disagreements between surface imaging and MV filming were also seen in the LOAs between postshift surfaces (i.e., at treatment position) and MV filmed treatment positions. Although the LOAs for postshift surfaces are overall smaller than for preshift surfaces, indicating that the MV film shifts moved the patient closer to the ideal treatment position, they remain larger than 8 mm, even when an AlignRT reference surface is used. This demonstrates that, while alignment based on bony anatomy may bring the overall position of the patient closer to the planned position, the breast tissue may not necessarily be closer to the planned position. This effect is exacerbated by breast deformation, as shown in Fig. 4. Depending upon the patient, differences in breast surface can range from significant and highly variable throughout treatment (see Patient B) to consistently minor (see Patient C). This variability might be amplified due to anatomical changes that patients may undergo from the time of simulation to the start of treatment. The patient's external anatomy can change throughout the treatment course due to factors such as healing from surgery, swelling from lymphatic drainage, weight fluctuations, or changes in the patient's comfort level in the treatment position. Surface imaging is more sensitive to these differences than conventional imaging since it analyzes the patient's topography to assess position.

The data presented here do not imply that the treatment position determined by one modality is more accurate than the other, but simply that each system provides different anatomical information. The importance of properly matching bony anatomy over external surface, or vice versa, should be determined by the physician based on the specific target and treatment technique for each patient. In addition to lower radiographic contrast with MV films, work published by Topolnjak et al.^(13)^ showed that registration with MV films could result in a 20%–50% underestimation of bony setup errors when compared to cone‐beam CT (CBCT). The authors postulated that the 2D nature of MV films limit their accuracy. Furthermore, Bert et al.^(7)^ also highlighted that MV images have a limited field‐of‐view, which typically does not include the patient's arms. Variations in arm location can affect the breast surface. In our experience, arm mispositioning leads to a CC offset when registering the breast surface, possibly causing the lack of correlation between MV and surface in the CC direction (see Table 2). Because surface imaging provides a 3D image that encompasses a larger view of the patient including the arms and hips, it may facilitate more accurate matching to the planned position.

Jain et al.^(4)^ studied the extent of interfraction motion by acquiring daily CBCT scans of ten breast cancer patients positioned based on focus‐to‐skin distances and MV films. They reported systematic/random errors of 2.8/3.5 mm along AP, 2.3/3.2 mm along CC, and 5.7/3.9 mm along LR directions. These values are comparable to the preshifts surface imaging errors. This is likely due to the fact that both surface imaging and CBCT provide therapists with a 3D representation of the soft tissue position. The advantage of surface imaging is that it provides real‐time feedback when adjusting the patient without the use of ionizing radiation. Conversely, CBCT allows for visualization of internal structures and clips only at the time of the scan and deposits extra dose to the patient. If both techniques yield similar patient positional accuracy, minimization of unnecessary dose to radiation‐sensitive organs, such as the contra–lateral breast, and instant feedback on patient position changes become key benefits of surface imaging.

One study directly investigated the use of surface imaging for whole breast patient positioning. Shah et al.^(5)^ compared setup with AlignRT to conventional alignment based on skin marks and portal imaging verification. In 14% of the cases when surface imaging was used for patient alignment and verified with MV films, disagreements greater than 3 mm were found. However, the magnitude of the discrepancy was not reported. Also, the dosimetric changes reported by Shah and colleagues did not account for any breast deformation, as no new CT scans were acquired. Clinical implementation of surface imaging differed from the current study in several ways. First, an AlignRT reference surface was acquired at the first treatment and used for alignment. Second, the ROI used for registration did not include the pendulous breast tissue. This may explain the smaller preshifts systematic/random errors of 2.6/3.2 mm along AP, 1.4/2.2 mm along CC, and 1.2/2.2 mm along LR.

When comparing daily alignment of the entire surface to the ${\text{DICOM}}_{\text{ref}}$, a large CC offset is seen. This is not surprising, as the patient's treatment position is determined by registering the MV films on the DRR. The DRR is created based on the 3 mm slice thickness planning CT scan; therefore, its resolution is the lowest along the CC direction. This leads to larger uncertainties in the patient's position in that direction. Using a surface image capture as the reference surface (${\text{AlignRT}}_{\text{ref}})$ resolves this discrepancy, as shown in Fig. 3. However, the limits of agreement are still large regardless of the reference surface used. Since the results of this study do not show a clear benefit of using one reference surface over the other, the use of ${\text{DICOM}}_{\text{ref}}$ might be preferred to minimize possible discrepancies from the planned position throughout the course of treatment.

The reproducibility of the arm position can be especially challenging from simulation to treatment delivery due to changes in the patient's comfort level in maintaining the treatment position for an extended period of time. Discrepancies in the positions of the arm and chin, which are not immobilized, often manifest in large CC shifts. An option to minimize the effect of the arms in the AlignRT deltas could be to create a new ROI including the torso, but excluding the arms and chin. Although the breast ROI inherently excludes them, a larger surface is needed to properly judge patient rotations.

While breast surface variability is not a new topic, the data presented in this work illustrate for the first time both quantitatively (through surface imaging analysis) and qualitatively (through comparisons between MV film and digitally reconstructed radiographs) the severity of pendulous tissue discrepancies from planning to treatment. This study also highlights the large inter‐ and intrapatient variability of the breast surface. These results indicate the importance of assessing the effects on treatment quality on a patient‐specific basis due to large differences in individuals' swelling, tissue mobility, etc. The influence of factors such as BMI and breast size on surface variations were not investigated in this study due to the limited number of subjects. However, the current patient cohort encompasses a wide range of BMI, making the results of this study applicable to a wide patient population.

The results of this work have led to two significant workflow changes in our clinic. First, orthogonal kV films are acquired with on‐board imaging (OBI) enabling simultaneous 2D‐matching of numerous anatomical landmarks distributed throughout the treatment field. This is in contrast to MV filming, in which individual landmarks are manually compared in a sequential manner. We believe the use of 2D matching focuses the efforts of the therapy team towards reproducing the simulated CT position. Second, the external patient surface is provided for each DRR, alerting the therapy team to discrepancies in the treated surface. We recommend the use of action thresholds to prevent treatment when large surface discrepancies occur. These thresholds should be guided by physician input and/or by collecting data on the dosimetric impact of these surface discrepancies.

## V. CONCLUSIONS

Breast tissue deformability poses a challenge when attempting to consistently position patients for radiotherapy. This study provides quantitative evidence that the use of bony alignment for setup can lead to large inter‐ and intrapatient variations in the breast surface position for patients with a range of body habitus, thereby affecting the tissue encompassed by the PTV between the two modalities. The low correlation between the MV and surface imaging shifts is attributed to the use of differing anatomical structures to determine the treatment position. Thus, the optimal anatomical structures to be used for patient alignment should be based on the best surrogate for the target location as indicated by the physician. Surface imaging, which provides direct information about the breast surface, should be considered a valuable addition to current positioning methods and incorporated into routine alignment protocols.

## ACKNOWLEDGMENTS

This work was presented in part at the American Association of Physicists in Medicine (AAPM) annual meeting in Indianapolis, IN in July 2013. Support for institutional review board approval was provided by the University of Chicago Comprehensive Cancer Center support grant P30 CA014599.

## Supporting information

Supplementary Material
